# Wnt10b Participates in Regulating Fatty Acid Synthesis in the Muscle of Zebrafish

**DOI:** 10.3390/cells8091011

**Published:** 2019-08-30

**Authors:** Dongwu Liu, Qiuxiang Pang, Qiang Han, Qilong Shi, Qin Zhang, Hairui Yu

**Affiliations:** 1 School of Agricultural Engineering and Food Science, Shandong University of Technology, Zibo 255049, China; 2 Anti-Aging & Regenerative Medicine Research Institution, School of Life Sciences, Shandong University of Technology, Zibo 255049, China; 3 Sunwin Biotech Shandong Co., Ltd., Weifang 262737, China; 4 Guangxi Key Laboratory for Polysaccharide Materials and Modifications, Guangxi Colleges and Universities Key Laboratory of Utilization of Microbial and Botanical Resources, School of Marine Science and Biotechnology, Guangxi University for Nationalities, Nanning 530008, China; 5 College of Biological and Agricultural Engineering, Weifang Bioengineering Technology Research Center, Weifang University, Weifang 261061, China

**Keywords:** Wnt10b, GSK-3β, β-catenin, lipid deposition, zebrafish

## Abstract

There are 19 Wnt genes in mammals that belong to 12 subfamilies. Wnt signaling pathways participate in regulating numerous homeostatic and developmental processes in animals. However, the function of Wnt10b in fatty acid synthesis remains unclear in fish species. In the present study, we uncovered the role of the Wnt10b signaling pathway in the regulation of fatty acid synthesis in the muscle of zebrafish. The gene of Wnt10b was overexpressed in the muscle of zebrafish using pEGFP-N1-Wnt10b vector injection, which significantly decreased the expression of glycogen synthase kinase 3β (GSK-3β), but increased the expression of β-catenin, peroxisome proliferators-activated receptor γ (PPARγ), and CCAAT/enhancer binding protein α (C/EBPα). Moreover, the activity and mRNA expression of key lipogenic enzymes ATP-citrate lyase (ACL), acetyl-CoA carboxylase (ACC) and fatty acid synthetase (FAS), and the content of non-esterified fatty acids (NEFA), total cholesterol (TC), and triglyceride (TG) were also significantly decreased. Furthermore, interference of the Wnt10b gene significantly inhibited the expression of β-catenin, PPARγ, and C/EBPα, but significantly induced the expression of GSK-3β, FAS, ACC, and ACL. The content of NEFA, TC, and TG as well as the activity of FAS, ACC, and ACL significantly increased. Thus, our results showed that Wnt10b participates in regulating fatty acid synthesis via β-catenin, C/EBPα and PPARγ in the muscle of zebrafish.

## 1. Introduction

Wnts have been extensively studied in vertebrate model organisms. The human and mouse genomes contain 19 different Wnt genes, which have been assigned to 12 subclasses (Wnt1 through Wnt11 and Wnt16) based on peptide sequence identity [1,2]. The subfamilies of Wnt genes are involved in the early stages of evolution. Indeed, it has been reported that 11 subfamilies of Wnts are identified in Cnidaria, a group splitting from the last common ancestor of Bilaterians [3]. In addition, Wnts participate in regulating a variety of key developmental and homeostatic processes in animals. Over the last two decades, the implication of Wnts in a wide array of signaling pathways has made Wnts the subject of intense investigation in the studies of cell biology.

In the canonical Wnt signaling, Wnts play a significant role and the defining event is the accumulation of β-catenin. When Wnt ligands are deficient, glycogen synthase kinase 3β (GSK-3β) phosphorylates β-catenin, which is further degraded by a destruction complex, including GSK-3β, adenomatous polyposis coli (APC), and Axin [4,5]. In contrast, the binding of Wnts to a receptor on the cell membrane promotes the depolymerization of the destruction complex, thus leading to the accumulation of β-catenin. Then β-catenin moves to the nucleus and regulates the target gene expression [4,5].

The function of Wnt signaling in metabolic diseases has been illustrated in various genetic research. It was found that obesity was associated with Wnt10b polymorphisms [6,7], and Wnt5b variants induced type 2 diabetes [8]. In addition, Wnts play a key role in regulating lipid accumulation and adipogenesis in the liver [9]. The signaling of Wnts is associated with liver metabolism and the development of fatty liver. As a central molecule, β-catenin is critical in regulating the oxidation of fatty acid, lipogenesis, and ketogenesis in lipid metabolism of liver [10].

As a model organism, zebrafish are widely used in the study of molecular biology. In the muscle of fish, lipid is easily stored up for energy utilization. Moreover, the role of the Wnt10b signaling pathway in the fatty acid synthesis process is still unknown in fish species. Thus, in the present study we detected whether Wnt10b was involved in regulating fatty acid synthesis in the muscle of zebrafish by overexpressing and interfering with the Wnt10b gene.

## 2. Materials and Methods

### 2.1. Animals and Experimental Conditions

The zebrafish (AB strain) were, on average, ~3.6 cm in length and approximately six months of age. Zebrafish were cultured at a light:dark photoperiod (12 h:12 h) in dechlorinated water (28 °C) in flow-through tanks. Zebrafish were fed twice daily with a commercial diet (Sanyou Beautification Feed Tech Co., Ltd.,Beijing, China) and acclimated for two weeks. All animal experiments were approved by Shandong University of Technology’s Institutional Animal Care Committee in accordance with the Guidelines for Proper Conduct of Animal Experiments (Science Council of China).

### 2.2. Construction of pEGFP-N1-Wnt10b Vector

Firstly, the transmembrane domain of the Wnt10b protein was analyzed with TMHMM Server v. 2.0 (http://www.cbs.dtu.dk/services/TMHMM/). The extracellular domain of Wnt10b (GenBank: AY182171.1) was cloned with the primers Wnt10b-F1 and Wnt10b-R1 (Table 1). Then, the extracellular domain of Wnt10b with the restriction enzyme sites was cloned with the primers Wnt10b-F2 and Wnt10b-R2 (with *Hind* III and *Kpn* I restriction enzyme sites) (Table 1). Finally, the fragment of Wnt10b was inserted into the plasmid pEGFP-N1 (Beijing TransGen Biotech Co. Ltd., Beijing, China). The construction of the pEGFP-N1-Wnt10b vector is shown in the Supplementary Material (Figure S1). This vector was designated as pEGFP-N1-Wnt10b and transformed into *E. coli* DH5a for plasmid amplification.

### 2.3. Injection of the pEGFP-N1-Wnt10b Vector

Forty-eight male zebrafish were randomly distributed into eight glass tanks. For the experimental group (4 tanks), according to the method of Hansen et al. [11], the vector of pEGFP-N1-Wnt10b (500 ng dissolved in 10 μL PBS) was injected into the dorsum muscle of each fish, and fish received one injection at day one. The same volume of PBS was injected into the muscle of each fish in the control group (4 tanks). Six days later, four independent muscle samples (each sample collected from three fish) were sampled from the experimental groups, which were used for molecular biology analysis. In addition, the other four muscle samples were collected for biochemical analysis. For the control group, the fish were sampled as the experimental group.

### 2.4. Wnt10b RNA Interference

The double-stranded RNA (dsRNA) was synthesized with the primers Wnt10b-F3 and Wnt10b-R3 according to the method of Arockiaraj et al. [12] (Table 1). Forty-eight male zebrafish were randomly distributed into eight glass tanks. For the experimental group (4 tanks), 200 ng dsRNA diluted in PBS (10 μL) was injected into the dorsum muscle of the fish. Fish received one injection at day one. The fish of the control group (4 tanks) received 10 μL PBS. Six days later, the muscles were sampled as the experiment of pEGFP-N1-Wnt10b vector treatment.

### 2.5. Real-Time Quantitative Polymerase Chain Reaction

Total RNA was extracted and transcribed to cDNA by using PrimeScript™ RT Reagent Kit (Takara, Japan). To detect the gene expression level, SYBR^®^ Premix Ex Taq™ II (Takara, Japan) was used. The primer sequences for Wnt10b, β-catenin, GSK-3β, C/EBPα, PPARγ, acetyl-CoA carboxylase (ACC), ATP-citrate lyase (ACL), fatty acid synthetase (FAS), HMG-CoA reductase (HMGCR), and reference gene (β-actin) were designed (Table 2). Roche LightCycler480^®^ II (Switzerland) was used to perform real-time PCR. The 2^−ΔΔCT^ method was used to analyze the level of gene expression, and β-actin as the reference gene [13].

### 2.6. Western-Blot Analysis

A protein Extraction Kit (Beyotime Biotechnology, Wuhan, China) was used to extract protein from muscle samples. After gel electrophoresis and blocking, the membranes were probed with the primary antibodies. Antibodies directed against GSK-3β (Cat. No. 12456, 1:1000), C/EBPα (Cat. No. 8178, 1:1000), β-catenin (Cat. No. 8480, 1:1000), and PPARγ (Cat. No. 2435, 1:1000) were obtained from Cell Signaling Technology Inc. Antibodies against Wnt10b (sc-7432, 1:500) and β-actin (sc-1615, 1:10,000) was purchased from Santa-Cruz Inc. Then the secondary HRP-conjugated antibody was used. Image J software (USA) was used to perform the densitometry analyses.

### 2.7. Biochemical Index Analysis

Cell lysates were homogenized and collected for biochemical analysis. The content of non-esterified fatty acids (NEFA), total cholesterol (TC), and triglyceride (TG) as well as the activity of FAS, ACC, and ACL were assayed according to the instructions of the TG and TC kits (Nanjing Jiancheng Bioengineering Institute, Nanjing, China), and FAS, ACC, and ACL activity kits (Zhuocai biology Co., Ltd., Shanghai, China). Finally, to determine the protein concentration, coomassie brilliant blue G250 staining was used.

### 2.8. Statistical Analysis

Data were presented as mean values ± standard error of mean (s.e.m), and SPSS 16.0 (SPSS Inc., 2005, USA) was used to perform the statistical analyses. The differences were analyzed using a student’s *t*-test analysis of independent samples at *p* < 0.05.

## 3. Results

### 3.1. Effect of Wnt10b Gene Overexpression on the Gene Expression of Wnt10b, GSK-3β, β-catenin, C/EBPα, and PPARγ

**Figure 1:**
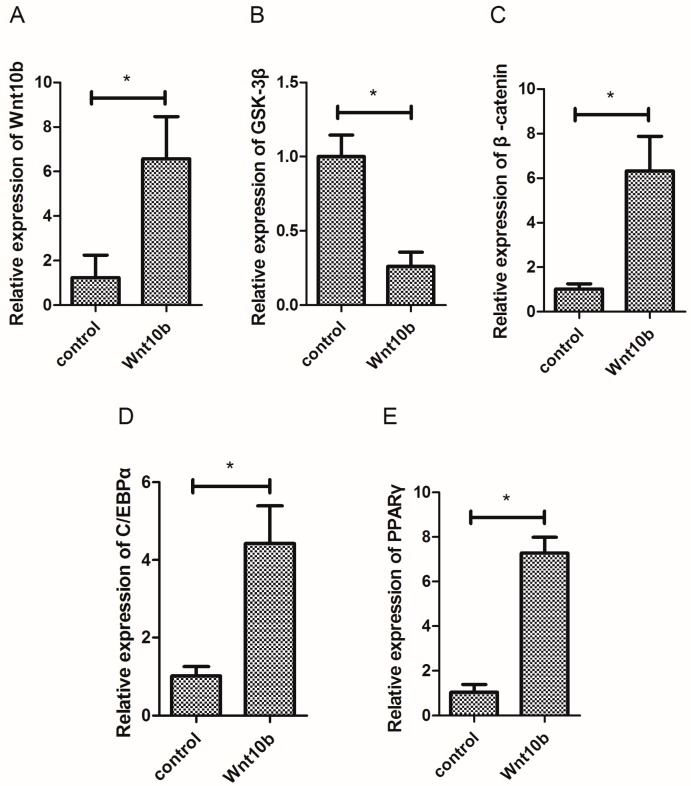
Effect of Wnt10b gene overexpression on the mRNA expression of Wnt10b, GSK-3β, β-catenin, C/EBPα, and PPARγ in the muscle of zebrafish. (**A**) Wnt10b; (**B**) GSK-3β; (**C**) β-catenin; (**D**) C/EBPα; (**E**) PPARγ. Values are expressed as means ± s.e.m. (*n* = 4). Statistically significant differences are denoted by an asterisk (* *p* < 0.05).

The overexpression of Wnt10b gene significantly induced the mRNA expression of Wnt10b and β-catenin (Figure 1A,C), but significantly decreased the mRNA expression of GSK-3β in the muscle of zebrafish (Figure 1B). Moreover, the overexpression of Wnt10b gene significantly increased the mRNA expression of C/EBPα and PPARγ (Figure 1D,E).

### 3.2. Effect of Wnt10b Gene Overexpression on the Protein Expression of Wnt10b, GSK-3β, β-catenin, C/EBPα, and PPARγ

**Figure 2:**
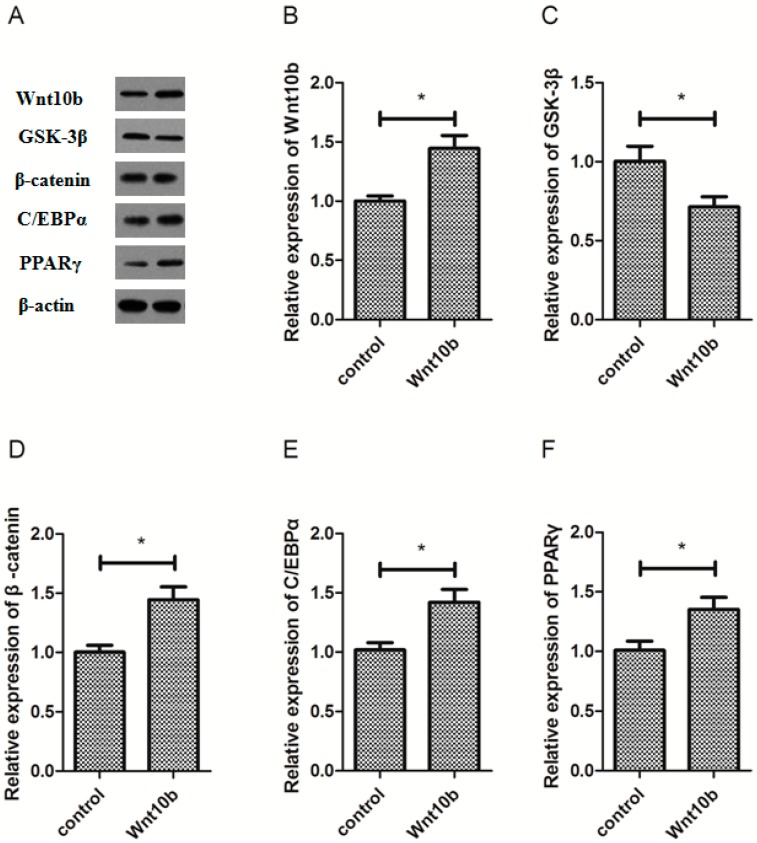
Effect of Wnt10b gene overexpression on the protein expression of Wnt10b, GSK-3β, β-catenin, C/EBPα, and PPARγ in the muscle of zebrafish. (**A**) The protein bands; (**B**) Wnt10b; (**C**) GSK-3β; (**D**) β-catenin; (**E**) C/EBPα; (**F**) PPARγ. Values are expressed as means ± s.e.m. (*n* = 4). Statistically significant differences are denoted by an asterisk (* *p* < 0.05).

The overexpression of Wnt10b gene significantly increased the protein expression of Wnt10b and β-catenin (Figure 2A,B,D), however, significantly inhibited the protein expression of GSK-3β in the muscle of zebrafish (Figure 2A,C). Nevertheless, the protein expression of C/EBPα and PPARγ was significantly induced by Wnt10b gene overexpression in the muscle of zebrafish (Figure 2A,E,F).

### 3.3. Effect of Wnt10b Gene Overexpression on the Gene Expression and Activity of Fatty Acid Synthetase (FAS), Acetyl-CoA Carboxylase (ACC) and ATP-Citrate Lyase (ACL),

**Figure 3:**
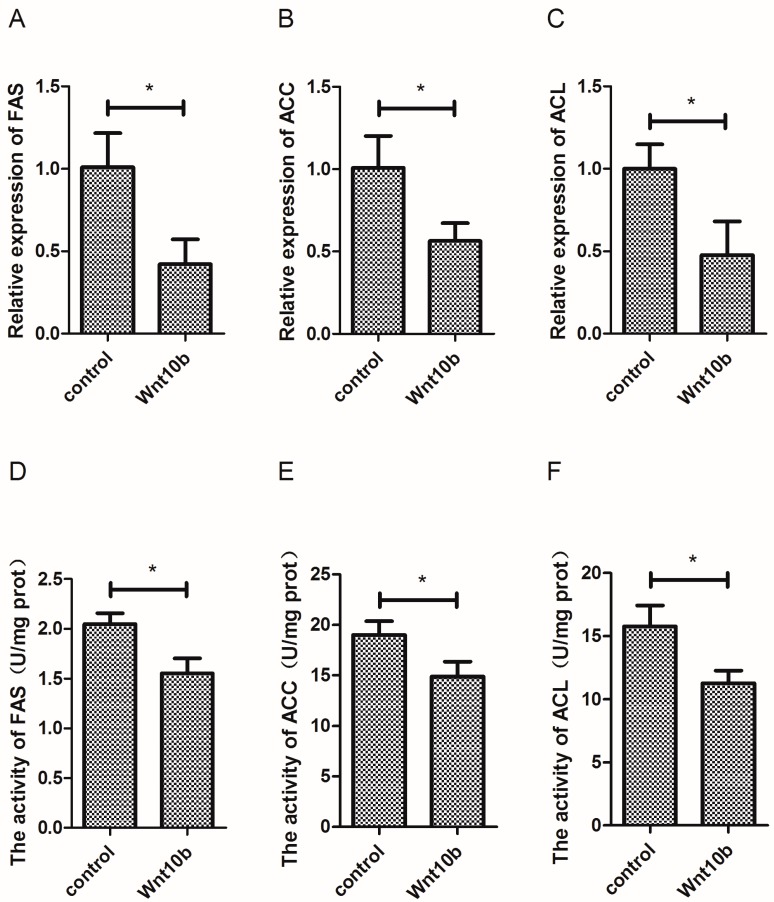
Effect of Wnt10b gene overexpression on the mRNA expression and activity of Fatty Acid Synthetase (FAS), Acetyl-CoA Carboxylase (ACC), and ATP-Citrate Lyase (ACL) in the muscle of zebrafish. (**A**) The mRNA expression of FAS; (**B**) The mRNA expression of ACC; (**C**) The mRNA expression of ACL; (**D**) The activity of FAS; (**E**) The activity of ACC; (**F**) The activity of ACL. Values are expressed as means ± s.e.m. (*n* = 4). Statistically significant differences are denoted by an asterisk (* *p* < 0.05).

As shown on Figure 3, Wnt10b gene overexpression significantly decreased the mRNA expression of FAS, ACC, and ACL (Figure 3A–C). Moreover, the activity of FAS, ACC, and ACL was also significantly decreased by Wnt10b gene overexpression in the muscle of zebrafish (Figure 3D–F).

### 3.4. Effect of Wnt10b Gene Overexpression on the Biochemical Index in the Muscle

**Figure 4:**
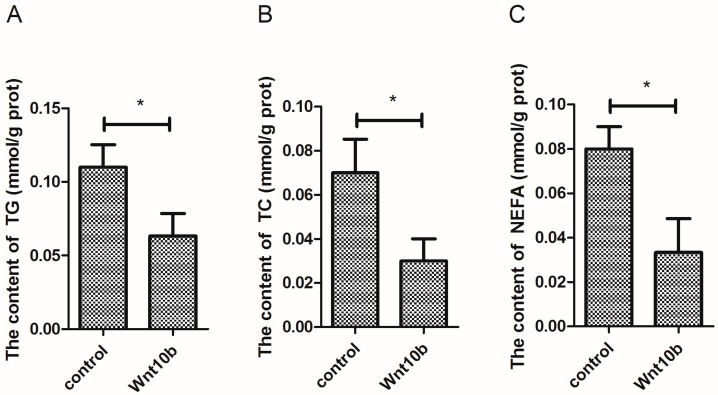
Effect of Wnt10b gene overexpression on the content of triglyceride (TG), total cholesterol (TC), and non-esterified fatty acids (NEFA) in the muscle. (**A**) TG; (**B**) TC; (**C**) NEFA. Values are expressed as means ± s.e.m. (*n* = 4). Statistically significant differences are denoted by an asterisk (* *p* < 0.05).

The content of TG and TC was significantly decreased by Wnt10b gene overexpression (Figure 4A,B). In addition, Wnt10b gene overexpression significantly decreased the content of NEFA in the muscle (Figure 4C).

### 3.5. Effect of Wnt10b Gene Interference on the Gene Expression of Wnt10b, GSK-3β, β-catenin, C/EBPα, and PPARγ

**Figure 5:**
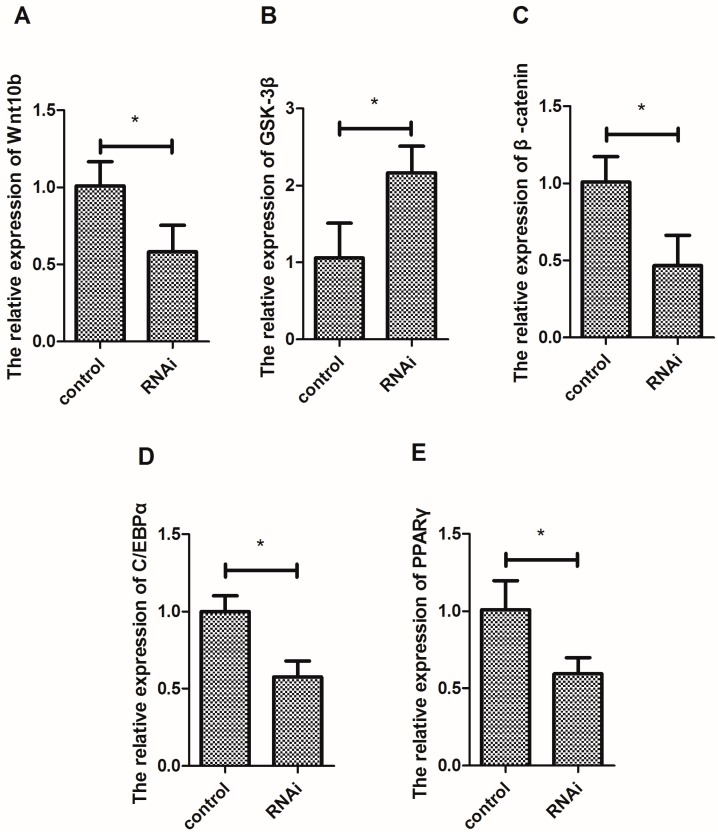
Effect of Wnt10b gene interference on the mRNA expression of Wnt10b, GSK-3β, β-catenin, C/EBPα, and PPARγ in the muscle of zebrafish. (**A**) Wnt10b; (**B**) GSK-3β; (**C**) β-catenin; (**D**) C/EBPα; (**E**) PPARγ. Values are expressed as means ± s.e.m. (*n* = 4). Statistically significant differences are denoted by an asterisk (* *p* < 0.05).

The interference of Wnt10b gene significantly decreased the mRNA expression of Wnt10b and β-catenin (Figure 5A,C), but significantly increased the expression of GSK-3β in the muscle (Figure 5B). Moreover, silencing Wnt10b gene significantly decreased the mRNA expression of C/EBPα and PPARγ (Figure 5D,E).

### 3.6. Effect of Wnt10b Gene Interference on the Protein Expression of Wnt10b, GSK-3β, β-catenin, C/EBPα, and PPARγ

**Figure 6:**
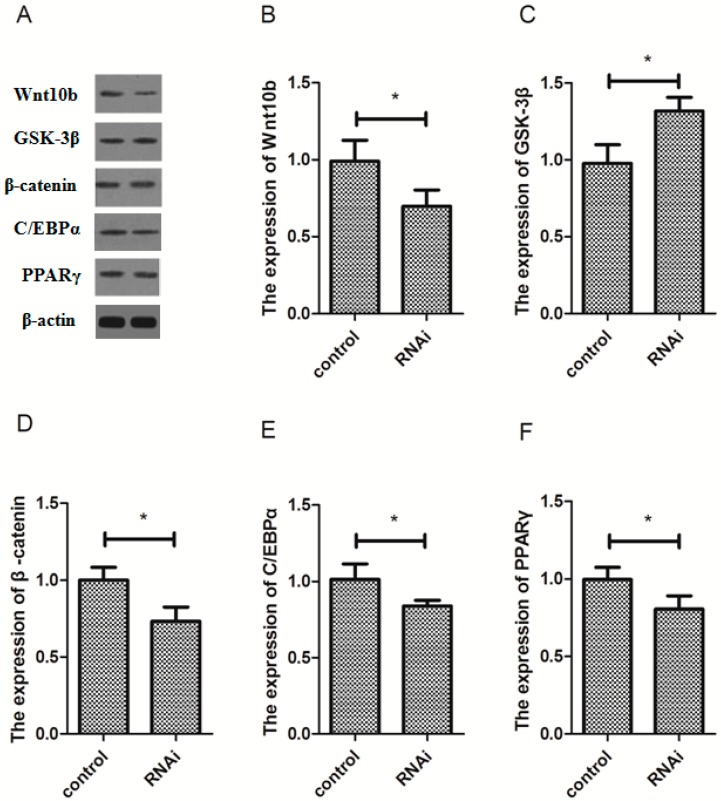
Effect of Wnt10b gene interference on the protein expression of Wnt10b, GSK-3β, β-catenin, C/EBPα, and PPARγ in the muscle of zebrafish. (**A**) The protein bands; (**B**) Wnt10b; (**C**) GSK-3β; (**D**) β-catenin; (**E**) C/EBPα; (**F**) PPARγ. Values are expressed as means ± s.e.m. (*n* = 4). Statistically significant differences are denoted by an asterisk (* *p* < 0.05).

The protein expression of Wnt10b and β-catenin was significantly decreased by Wnt10b gene interference in the muscle (Figure 6A,B,D). However, Wnt10b gene interference significantly increased the protein expression of GSK-3β (Figure 6A,C). Nevertheless, the protein expression of C/EBPα and PPARγ was significantly inhibited by Wnt10b gene interference in the muscle of zebrafish (Figure 6A,E,F).

### 3.7. Effect of Wnt10b Gene Interference on the Gene Expression and Activity of FAS, ACC, and ACL

**Figure 7:**
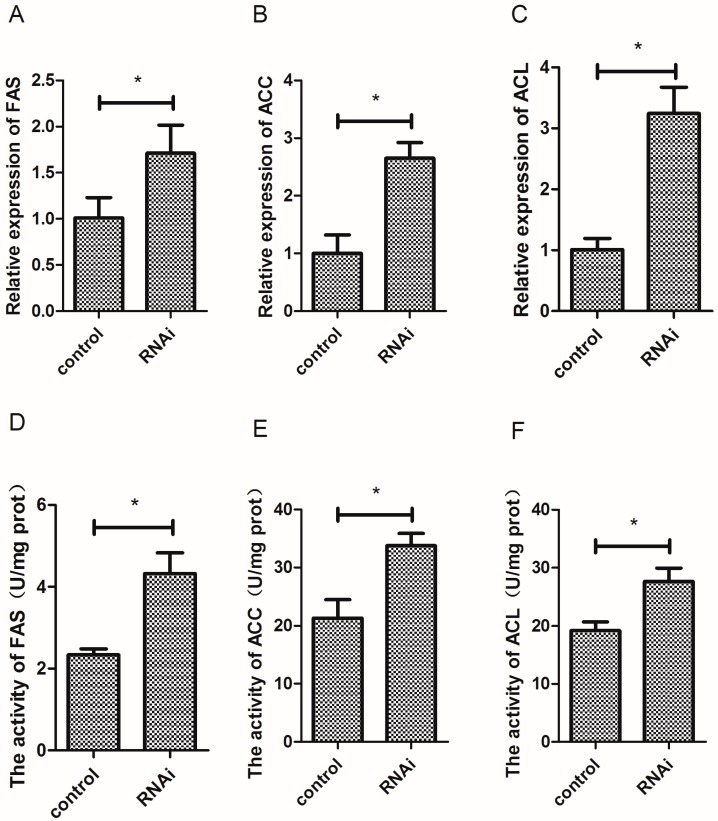
Effect of Wnt10b gene interference on the mRNA expression and activity of Fatty Acid Synthetase (FAS), Acetyl-CoA Carboxylase (ACC), and ATP-Citrate Lyase (ACL) in the muscle of zebrafish. (**A**) The mRNA expression of FAS; (**B**) The mRNA expression of ACC; (**C**) The mRNA expression of ACL; (**D**) The activity of FAS; (**E**) The activity of ACC; (**F**) The activity of ACL. Values are expressed as means ± s.e.m. (*n* = 4). Statistically significant differences are denoted by an asterisk (* *p* < 0.05).

Wnt10b gene interference significantly increased the mRNA expression of FAS, ACC, and ACL (Figure 7A–C). The activity of FAS, ACC, and ACL was also significantly increased by Wnt10b gene interference in the muscle of zebrafish (Figure 7D–F).

### 3.8. Effect of Wnt10b Gene Interference on the Biochemical Index in the Muscle

**Figure 8:**
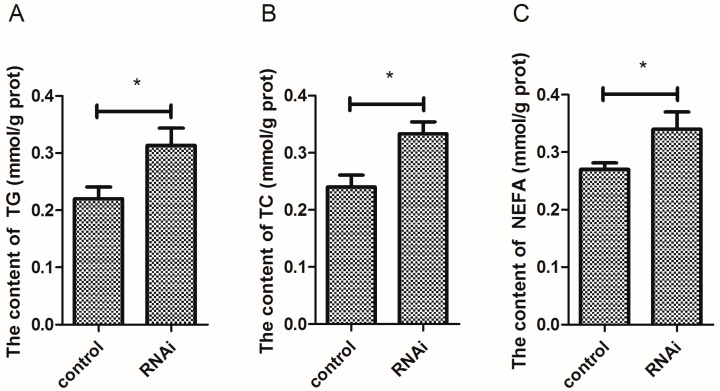
Effect of Wnt10b gene interference on the content of triglyceride (TG), total cholesterol (TC), and non-esterified fatty acids (NEFA) in the muscle. (**A**) TG; (**B**) TC; (**C**) NEFA. Values are expressed as means ± s.e.m. (*n* = 4). Statistically significant differences are denoted by an asterisk (* *p* < 0.05).

The content of TG and TC was significantly increased by Wnt10b gene interference (Figure 8A,B). In addition, Wnt10b gene interference significantly increased the content of NEFA in the muscle (Figure 8C).

### 3.9. Effect of Wnt10b Gene Overexpression and Interference on the mRNA Expression of HMGCR in the Muscle of Zebrafish

**Figure 9:**
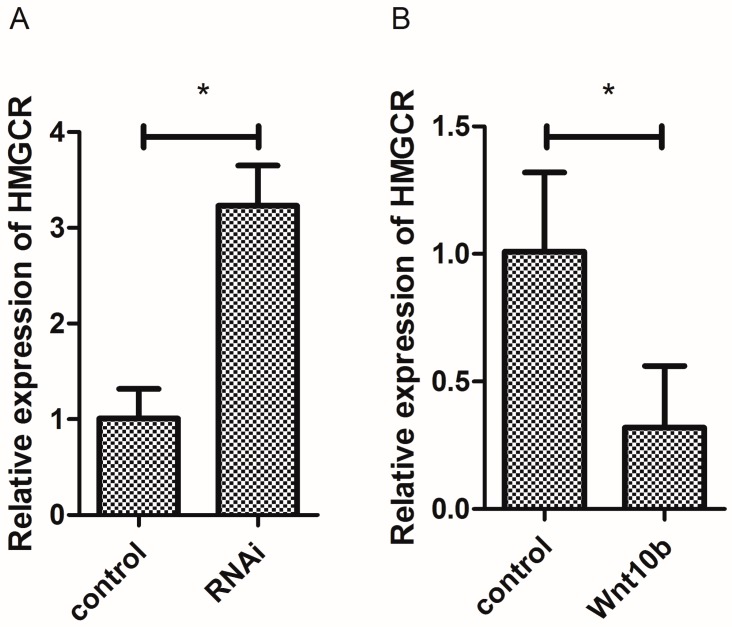
Effect of Wnt10b gene interference and overexpression on the mRNA expression of HMG-CoA reductase (HMGCR) in the muscle of zebrafish. (**A**) Effect of Wnt10b gene interference on the mRNA expression of HMGCR; (**B**) Effect of Wnt10b gene overexpression on the mRNA expression of HMGCR. Values are expressed as means ± s.e.m. (*n* = 4). Statistically significant differences are denoted by an asterisk (* *p* < 0.05).

Wnt10b gene interference significantly induced the mRNA expression of HMGCR, while Wnt10b gene overexpression significantly decreased the expression of HMGCR (Figure 9A,B).

## 4. Discussion

Wnts exerts functions through multiple effectors, among which β-catenin is the most important one [4,5,14,15]. As Wnt ligands bound to receptors on the cell membrane, GSK-3β activity was impeded and β-catenin accumulated [4,16,17]. In addition, GSK-3β can phosphorylate β-catenin, which leads to the degradation of β-catenin [18,19]. In the present study, the overexpression of Wnt10b gene decreased GSK-3β expression and increased β-catenin expression in the muscle, whereas the interference of Wnt10b gene exhibited a negative effect on the expression of GSK-3β and β-catenin. Thus, the results indicated that Wnt10b induced β-catenin signaling by inhibiting GSK-3β expression.

In the muscle of fish, lipid is easily stored up for energy utilization [20,21,22]. As main lipogenic enzymes, FAS, ACC, and ACL play a key role in lipid deposition in the muscle of fish. In the present study, the activity and mRNA expression of ACC, FAS, and ACL in muscle were significantly decreased by Wnt10b gene overexpression, but significantly induced by Wnt10b gene interference. These results indicate that Wnt signaling may inhibit the synthesis of fatty acid through decreasing FAS, ACC, and ACL activity. In addition, the content of NEFA, TC, and TG was significantly decreased by Wnt10b gene overexpression in the muscle of zebrafish, but significantly increased by Wnt10b gene interference. In the process of lipid homeostasis, a balance between the synthesis and hydrolysis of fatty acid is present in the normal physiological status of a cell. Since the biochemical indexes were significantly affected by Wnt10b, Wnt10b signaling may have regulated the levels of NEFA, TC, and TG through regulating ACC, FAS, and ACL activity in the muscle of the zebrafish.

As a rate-limiting enzyme in the process of cholesterol synthesis, the activity and expression of HMGCR is regulated by various small molecular metabolites. The expression level of HMGCR is closely related to the content of TC. In this study, the overexpression of the Wnt10b gene significantly inhibited the mRNA expression of HMGCR, but Wnt10b gene interference significantly induced HMGCR expression. The decrease of HMGCR expression induced by Wnt10b gene overexpression may result in the decrease of TC content in the muscle of zebrafish.

The development of adipose tissue comprises the proliferation of new adipocytes as well as the hypertrophy of existing adipocytes. It has been observed that PPARγ and C/EBPα participate in regulating the expression of genes related to the adipocyte proliferation [23]. In the process of adipocyte differentiation, a variety of proteins and enzymes on the lipid synthesis were regulated by PPARγ and C/EBPα [24,25]. Furthermore, Wnt/β-catenin signaling regulates the expression level of PPARγ and C/EBPα [26,27], which is closely related to the process of lipid deposition [27]. Therefore, the expression levels of PPARγ and C/EBPα were investigated in the present study. Our data showed that the expression of C/EBPα and PPARγ was induced by Wnt10b gene overexpression and inhibited by silencing of the Wnt10b gene. This shows that C/EBPα and PPARγ are involved in the process of fatty acid synthesis. However, whether Wnt10b gene participates in regulating PPARβ and PPARδ needs to be researched in the future.

In summary, we explored a novel mechanism that Wnt10b participates in regulating fatty acid synthesis in the muscle of zebrafish. We modulated innate Wnt10b levels by gene overexpression or interference, and our results revealed that Wnt10b induced GSK-3β/β-catenin signaling, which further inhibited fatty acid synthesis in the muscle of zebrafish.

## Figures and Tables

**Table 1:** Sequence of the primers used in this study.

Primer	Sequence (5′-3′)	Direction
Wnt10b-F1	CAATGACATCCTCGGCCTGAAG	Forward
Wnt10b-R1	TCACTTGCACACATTAACCCACTC	Reverse
Wnt10b-F2	CCCAAGCTTGCCACCATGAAGGTGGCAGGAGAGCCAGTGC	Forward
Wnt10b-R2	CGGGGTACCCCCTTGCACACATTAACCCACTCTG	Reverse
Wnt10b-F3	GATCACTAATACGACTCACTATAGGGGAGACCAGCGCTGGAACTGCTC	Forward
Wnt10b-R3	GATCACTAATACGACTCACTATAGGGCCACTCTGTT ATTACGAATCC	Reverse

Restriction enzyme sites (*Hind* III and *Kpn* I) are underlined.

**Table 2:** Real-time quantitative Polymerase Chain Reaction primers for genes of zebrafish.

Target Gene	Forward (5′-3′)	Reverse (5′-3′)	GenBank
Wnt10b	TCCTGAAACAGGCTCGAAGT	GCTGCTCACTTGCACACATT	AY182171.1
GSK-3β	TCTGCTCACCGTTTCCTTTC	CTCCGACCCACTTAACTCCA	NM_131381.1
β-catenin	GGAGCTCACCAGCTCTCTGT	TAGCTTGGGTCGTCCTGTCT	NM_001001889.1
C/EBPα	CACAACAGCTCCAAGCAAGA	AATCCATGTAGCCGTTCAGG	BC063934.1
PPARγ	CTGGACATCAAGCCCTTCTC	AGCTGTACATGTGCGTCAGG	NM_131467.1
FAS	ACAATGCTGGTGACAGTGGA	TACGTGTGGGCAGTCTCAAG	XM_009306806.2
ACC	AGGTGGTACGGATGGCTGCTC	GACGGTGCTGGACGCTGTTG	NM_001271308.1
ACL	AGACCTGATCTCCAGCCTCACATC	ATGCCACTGTCGAATGCCTTACTG	BC076484.1
HMGCR	ACGTCATCGGTTACATGCCAGTTC	GCCTTCAGTTGTCGCCATCGG	NM_001079977.2
β-actin	CCGTGACATCAAGGAGAAGC	TACCGCAAGATTCCATACCC	AF057040.1

## Supplementary Materials

The following are available online at https://www.mdpi.com/2073-4409/8/9/1011/s1, Figure S1: The construction of pEGFP-N1-Wnt10b.
