# Supplementary material for: Wnt10b Participates in Regulating Fatty Acid Synthesis in the Muscle of Zebrafish
Source: Cells. 2019 Aug 30;8(9):1011. doi: 10.3390/cells8091011 (PMC6769891; doi:10.3390/cells8091011)
Supplement: Supplementary file 1 [file cells-08-01011-s001.pdf]

Supplementary material

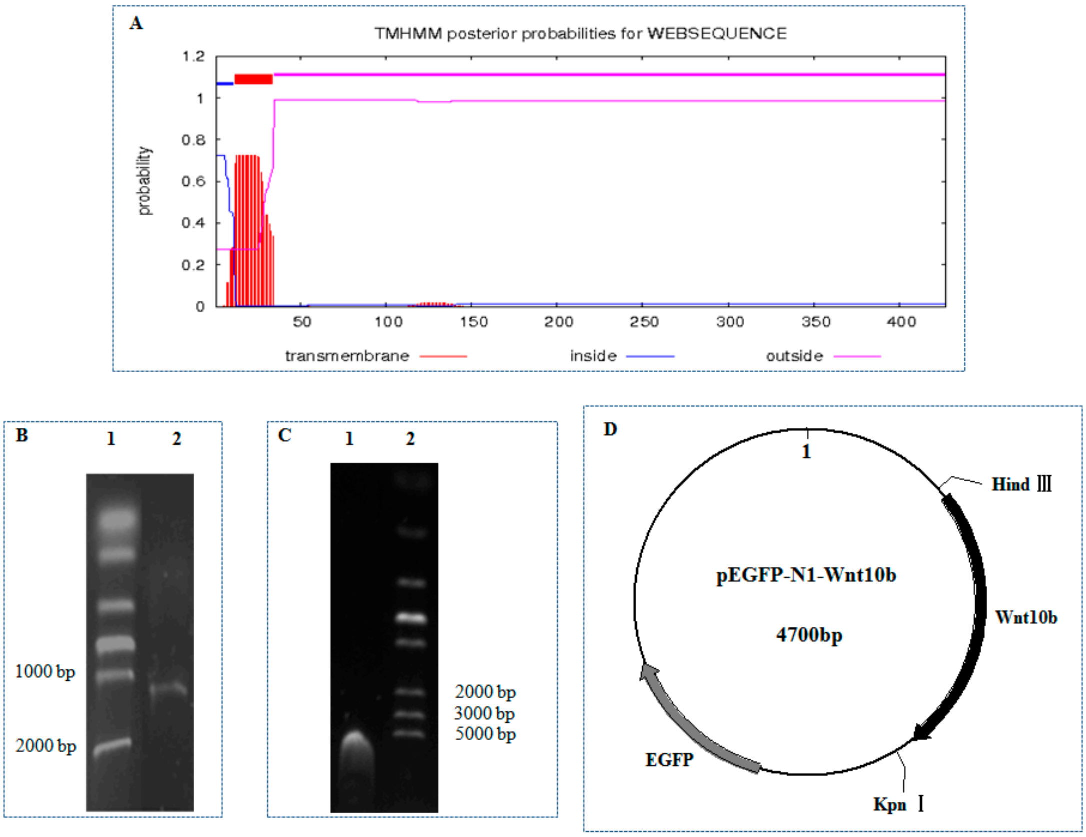

Figure S1. The construction of pEGFP-N1-Wnt10b.

(A) Transmembrane domain analysis of Wnt10b protein.

(B) The extracellular domain of Wnt10b was cloned according to the primers Wnt10b-F1 and Wnt10b-R1. Lane 1, DNA marker; Lane 2, Wnt10b.

(C) The connected fragment of Wnt10b and pEGFP-N1 was amplified using PCR. Lane 1, the connected fragment of Wnt10b and pEGFP-N1; Lane 2, DNA marker.

(D) The map of pEGFP-N1-Wnt10b.
